# Physiologic Effects of Isolated or Synthetic Dietary Fiber in Children: A Scoping Review

**DOI:** 10.1016/j.cdnut.2023.102074

**Published:** 2024-01-04

**Authors:** Linfei Chen, Esther A Avendano, Angelica Valdes-Valderrama, Jessie L Lan, Dominique Tye, Rebecca A Morin, Kara A Staffier, Nicola M McKeown, Nanguneri Nirmala

**Affiliations:** 1 Tufts University School of Medicine, Boston, MA, United States; 2 Institute for Clinical Research and Health Policy Studies, Center for Clinical Evidence Synthesis, Tufts Medical Center, Boston, MA, United States; 3 Friedman School of Nutrition Science and Policy, Tufts University, Boston, MA, United States; 4 Hirsh Health Sciences Library, Tufts University, Boston, MA, United States; 5 American College of Life Style Medicine, Chesterfield, MO, United States; 6 Department of Health Sciences, Sargent College of Health and Rehabilitation Sciences, Boston University, Boston, MA, United States

**Keywords:** dietary fiber, isolated fiber, synthetic fiber, child health, scoping review, inulin-type fructans, psyllium

## Abstract

**Background:**

Fiber is an integral part of a healthy diet. Studies have shown that the fiber intake in children is below adequate amounts, leading to adverse health outcomes.

**Objectives:**

This study aimed to perform a scoping review to assess the available evidence for the impact of isolated and synthetic dietary fiber on children’s health outcomes.

**Methods:**

A systematic literature search was conducted in Ovid Medline, Ovid Global Health, Embase, and Cochrane Library via Wiley to identify randomized controlled trials (RCTs) in healthy children aged 1–18 y at baseline who consumed added, isolated, or synthetic dietary fiber. The outcomes of interest were categorized based on the Food and Drug Administration’s guidance for industry on nondigestible carbohydrates and the Vahouny Fiber Symposium criteria, which included reduced fasting blood, glucose, total and/or LDL cholesterol concentrations, attenuation of postprandial glycemia/insulinemia, increased fecal bulk/laxation, reduced transit time, weight loss/reduction in adiposity, reduced energy intake from food consumption, increased satiety, bone health/enhanced mineral absorption, and blood pressure. We also cataloged additional reported outcomes.

**Results:**

Of 3837 randomized controlled parallel or crossover trials screened at the abstract level, 160 were eligible for full-text review, and 32 included for data extraction. This scoping review presents analysis of data from 32 RCTs in children who were healthy, overweight/obese or had mild hypercholesterolemia. Inulin-type fructans (41%) and psyllium (22%) were the most frequently administered fiber types, with weight/adiposity, markers of lipid metabolism (41%), and bone-related markers (38%) being the most frequently reported health outcomes. Only a few RCTs have investigated the effects of laxation (9%), and none specifically studied the impact of fiber on reducing postprandial glycemia/insulinemia.

**Conclusions:**

This scoping review demonstrates sufficient evidence for conducting systematic reviews and meta-analyses for several outcomes. Evidence gaps remain on the impact of isolated fibers on outcomes such as laxation, colonic transit time, and postprandial glycemia/insulinemia in children.

## Introduction

In the United States, increasing rates of obesity and metabolic dysregulation are occurring in children. The prevalence of children who are overweight and obese has risen from 4% in 1975 [1] to 19.7% in 2020, which translates to an estimated 14.7 million children [2,3]. This rise is paralleled by an increase in associated cardiometabolic comorbidities, potentially predisposing youth to a lifetime of poor cardiometabolic health [4]. Globally, adherence to dietary recommendations is low [5], and although a dietary pattern high in fiber is linked to improved health [6], the majority of both United States adults and children fail to meet the recommended intakes [7]. Yet, research to support dietary recommendations for fiber intake in children is limited [8]. Currently, no dietary fiber intake recommendation exists for infants younger than 2 y [9,10], although the recommendation based on the American Heart Foundation for children older than 2 y is to increase dietary fiber to an amount equal to or greater than their age, plus 5 g [11], which may be insufficient to deliver health benefits. In the most recent 2020–2025 Dietary Guidelines for Americans, the adequate intakes for dietary fiber are based on the adult data showing fiber intake of 14 g/1000 kcal limits risk of coronary artery disease [12], with estimated ranges from 14 g/d (ages 2–3 y) to 25 g/d (ages 14–18 y) [13].

Natural sources of dietary fiber found in plant foods (ie, whole grains, vegetables, fruits, and legumes) have been linked with lower risk of developing cardiovascular disease, diabetes, and cancer in adults [[14], [15], [16]]. However, despite the widespread availability of fiber in foods and recognized health benefits, on average, adults consume ∼15 g of fiber per day [17], substantially lower than the recommended 22–34 g/d [13]. With low intakes among United States adults, it is not surprising that dietary fiber intake is inadequate among children, with intakes varying by age, ethnicity, and family income level [18,19]. Global intakes of dietary fiber in children (aged 2–5 y) range from 10 to 12 g/d, with higher ranges observed in older children and adolescents of 13–15 g/d [20].

Dietary fiber is not a single entity; different fiber types are found in plant foods, and fibers can be added to foods as ingredients, with each type delivering a variety of health benefits [6]. The 2009 Codex Alimentarius Commission definition of dietary fiber [21] is “carbohydrate polymers with 10 or more monomeric units that are not hydrolyzed by endogenous enzymes in the human small intestine.” Within this definition, dietary fiber can be categorized as the edible carbohydrate polymers naturally occurring in food; the fibers isolated from food raw material by physical, enzymatic, or chemical means; or fibers synthetically derived from carbohydrate polymers. With the exception of fiber naturally occurring in food, isolated or synthetic fibers (also referred to as added or functional fibers in earlier publications) must demonstrate a physiologic effect or benefit to health. However, dietary fiber recommendations do not indicate which fiber types or sources are needed to support specific physiologic health benefits. A better understanding of the health benefits of isolated and synthetic fibers is required to inform recommendations for fiber intake in children.

Socioeconomic status impacts dietary fiber intake as observed in a nationwide cross-sectional survey of caregivers designed to assess food and nutrient intakes of United States infants and children [8]. In addition, fiber intake from whole grains, fruits, and vegetables may be influenced by the food avoidance by some children [[22], [23], [24]]. Although the current recommendation is to obtain fiber from natural sources [25], foods with added fibers (isolated/synthetic) may be one way of increasing fiber intake in children, thus lessening the gap in fiber intake in children.

To date, few reviews have summarized the research on the impact of isolated or synthetic fiber on children’s health outcomes. Thus, we conducted a scoping review, a type of evidence synthesis that follows a systematic approach to provide an overview or map of the key concepts in a field of research [26]. This approach is useful for identifying themes such as theories, sources, and the conceptual boundaries of a topic, therefore pointing out remaining knowledge gaps [26,27].

This scoping review followed current published guidance [26,28] by defining PCC (population, concept, and context) for the review and mapping them to PICO (population, intervention, comparison, and outcomes) criteria [29] for future use in a systematic review. The questions that guided the objective of this scoping review were as follows: *1*) what is the body of published evidence regarding the effect of isolated and synthetic fiber on health outcomes in healthy children and adolescents? *2*) From the identified outcomes in the scoping review, what are the gaps in the research, and what outcomes could be identified for further research recommendations in this area? This scoping review aimed to inform both current evidence and future research on the effect of isolated and synthetic fibers in the diets of children.

## Methods

For this scoping review, we made the decision to conduct a broad search so that we could comprehensively capture the studies of the impact of fiber intake on children owing to limited available research in children. At the end of the scoping review, when systematic reviews are conducted, the scope can be narrowed to selective outcomes of interest. The strategy adopted to consolidate the evidence for the impact of isolated or synthetic fiber (context and concept) in children (population) is given below.

### Strategy of systematic search and study selection

#### Search databases for evidence collection

A strategy to ensure a systematic and comprehensive search was developed, and the scientific criteria and approach to selecting the studies were defined (Supplemental Table 1). The electronic search was conducted in November 2021 for randomized controlled intervention studies [randomized controlled trials (RCTs)], which reported isolated or synthetic fiber supplementation in children aged 1 to ≤18 y, published from 1946 to November 2021. The keywords for fiber were defined according to the Dietary Fibers and Human Health Outcomes Database [30] and further refined with the database developers’ expertise to meet this project’s requirements. In brief, the search strategy for Ovid Medline (Supplemental Table 1) was translated for the following databases: Ovid Medline Epub Ahead of Print, In-Process, In-Data-Review and Other Nonindexed Citations and Daily (2017 to November 05, 2021); Ovid Global Health (1910–2021 week 44); Embase; and Cochrane Library via Wiley, including results from CENTRAL. Additionally, a grey literature search was performed targeting relevant work in progress within the past 5 y. It included searches within publications from the Vahouny conferences [31], the United States National Library of Medicine clinical trials database (clinicaltrials.gov, 2022), and the United States Federal Science database (science.gov). Duplicate publications were removed using EndNote, and unique publication abstracts were uploaded into Covidence [32] for screening. Covidence is a web-based collaboration software platform that streamlines the production of systematic and other literature reviews, allowing for both screening and data extraction.

#### Population

Our scoping review was conducted on research studies in healthy children, which included those with overweight, obesity, or hypercholesterolemia. Further details on the eligibility criteria are reported in Table 1.

**TABLE 1:** Inclusion and exclusion criteria for the scoping review

Inclusion criteria	
Study design	Randomized controlled trials
Study population	Children and teenagers aged between 1 and 18 y
Health status at baseline	Healthy weight, overweight, obese, hypercholesterolemia
Exposure	Isolated or synthetic dietary fiber
Outcome categories	Anthropometric parameters; bioavailability of minerals; bone-related outcomes; colonic fermentation; eating behaviors; fecal bulk/laxation; gastrointestinal symptoms; glucose and insulin metabolism; lipids; markers of immune function; markers of inflammation; modulation of colonic microflora; reduced energy intake from food consumption; satiety; weight/adiposity
Exclusion criteria	
Study design	Nonrandomized controlled trials
	Cross-sectional studies
	Literature reviews
	Case reports
	Case–control
	Prospective and retrospective cohort
Exposure	Fiber from fruits and vegetables
	Infant formula
Study population	Infants younger than 1 y
	Adults older than 18 y

#### Intervention

Included studies were RCTs administering a broad spectrum of isolated and synthetic fiber. Fiber names/descriptions were extracted into the database as described in the publication; however, for analysis purposes, we grouped the fibers into broad categories.

#### Outcomes

Based on the Food and Drug Administration’s (FDA) guidance for industry on physiologic effects of isolated or synthetic [33] nondigestible carbohydrates, the following 8 outcomes were considered in our review: *1*) fasting blood total and/or LDL cholesterol concentrations, *2*) attenuation of postprandial glycemia/insulinemia, *3*) fecal bulk/laxation, *4*) colonic transit time, *5*) weight loss/reduction in adiposity, *6*) energy intake from food consumption, *7*) satiety, and *8*) bone health/enhanced mineral absorption. In addition, based on the Vahouny Fiber Symposium criteria [31], the following Vahouny outcomes were distinct from FDA-recommended outcomes and include the following: *1*) blood pressure, *2*) modulation of colonic microflora, and *3*) colonic fermentation/short-chain fatty acid production. Finally, other lipid-related outcomes such as HDL cholesterol, triglycerides (TGs), and apolipoproteins; other measures of glucose and insulin metabolism (HbA1c and C-peptide), and gastrointestinal (GI)-related symptoms were also extracted from the publications. In addition, we also extracted outcomes outside these categories, and classified them as “other” outcomes (Table 2). These mainly include the following: *1*) bioavailability of minerals, *2*) glucose and insulin metabolism, *3*) anthropometric parameters, *4*) markers of inflammation and immune function, and *5*) eating behaviors.

**TABLE 2:** Number of studies per outcome group

Source	Outcomes	Number of Studies
FDA/Vahouny	Weight/adiposity	13
FDA/Vahouny	Bone-related outcomes (including calcium absorption and retention)	12
FDA/Vahouny	Total and LDL cholesterol	10
FDA/Vahouny	Fecal bulk/laxation	3
FDA/Vahouny	Satiety	4
FDA/Vahouny	Postprandial glycemia/insulinemia	0
FDA/Vahouny	Transit time	0
FDA	Reduced energy intake from food consumption	5
Vahouny	Blood pressure	5
Vahouny	Colonic fermentation/short-chain fatty acid production	3
Vahouny	Modulation of colonic microflora	6
Vahouny: Other	Lipids (HDL, TG, and apolipoproteins)	13
Vahouny: Other	GI symptoms	5
Other	Bioavailability of minerals	7
Other	Glucose and insulin metabolism	7
Other	Anthropometric parameters	7
Other	Markers of inflammation and immune function	5
Other	Eating behaviors	3

Abbreviations: GI, gastrointestinal; TG, triglyceride.

The biochemical outcomes were assessed using objective measures like standard assays (for example, lipids and glucose). Some outcomes were assessed using subjective measures. In the studies that reported satiety, this outcome was assessed using subjective measures of satiety (Children’s eating Behavior Questionnaire, appetite rating questionnaires, and satiety efficiency index). Change in energy intakes were captured using self-reported intakes or weighed food records. Subjective measures, based on questionnaires, were also used to capture eating and other behavioral outcomes.

With respect to bone-related outcomes, the following were included in this category: bone mineral content; bone mineral density (BMD); biochemical markers of bone dynamics, that is, markers of bone formation (osteocalcin) or bone resorption (pyridinoline and deoxypyridinoline); hormones related to calcium metabolism [for example, intact parathyroid hormone 25(OH) vitamin D and free vitamin D index], calcium absorption, retention and excretion, and magnesium absorption.

#### Article screening

Two reviewers independently screened each abstract identified in the literature search for inclusion in the review. Articles were tagged as included or excluded. When the 2 reviewers differed in their article assessment, these conflicts were resolved during weekly meetings with the research team. Abstracts meeting the initial screening criteria were moved to the full-text screening stage. The full article texts were then screened in the same manner. Studies that met the inclusion criteria during full-text screening were accepted for inclusion and data extracted.

### Data extraction and synthesis

A data extraction template was created within the Covidence software application to extract data, including publication year, study country, study design, blinding, study duration, funding sources, possible conflicts of interest, recruitment criteria, method of recruitment, number of participants, baseline population characteristics, information about the fiber used for the intervention, and health outcomes studied. For each study, the data were extracted individually by 2 team members. A third team member resolved the conflicts for the fields that were not identical in the 2 extractions. The extracted data were downloaded into Microsoft Excel 2022, analyzed, and summarized according to the outcome and fiber type. The program R [34] was used to create tables and figures shown in the results section. The data extracted included the PICO criteria, as described in Table 3.

**TABLE 3:** PICO criteria for data extraction

PICO criteria	Details
Population (P)	Age range, baseline health, country
Intervention (I)	Trial design, duration, nature of the fiber, (brand name products and their component ingredients), and dosage of fiber
Comparator (C)	Details of the fiber equivalent in the comparator arm
Outcome (O)	Type of outcome and effect of fiber on outcome (its statistical significance)

## Results

### Search summary

**FIGURE 1:**
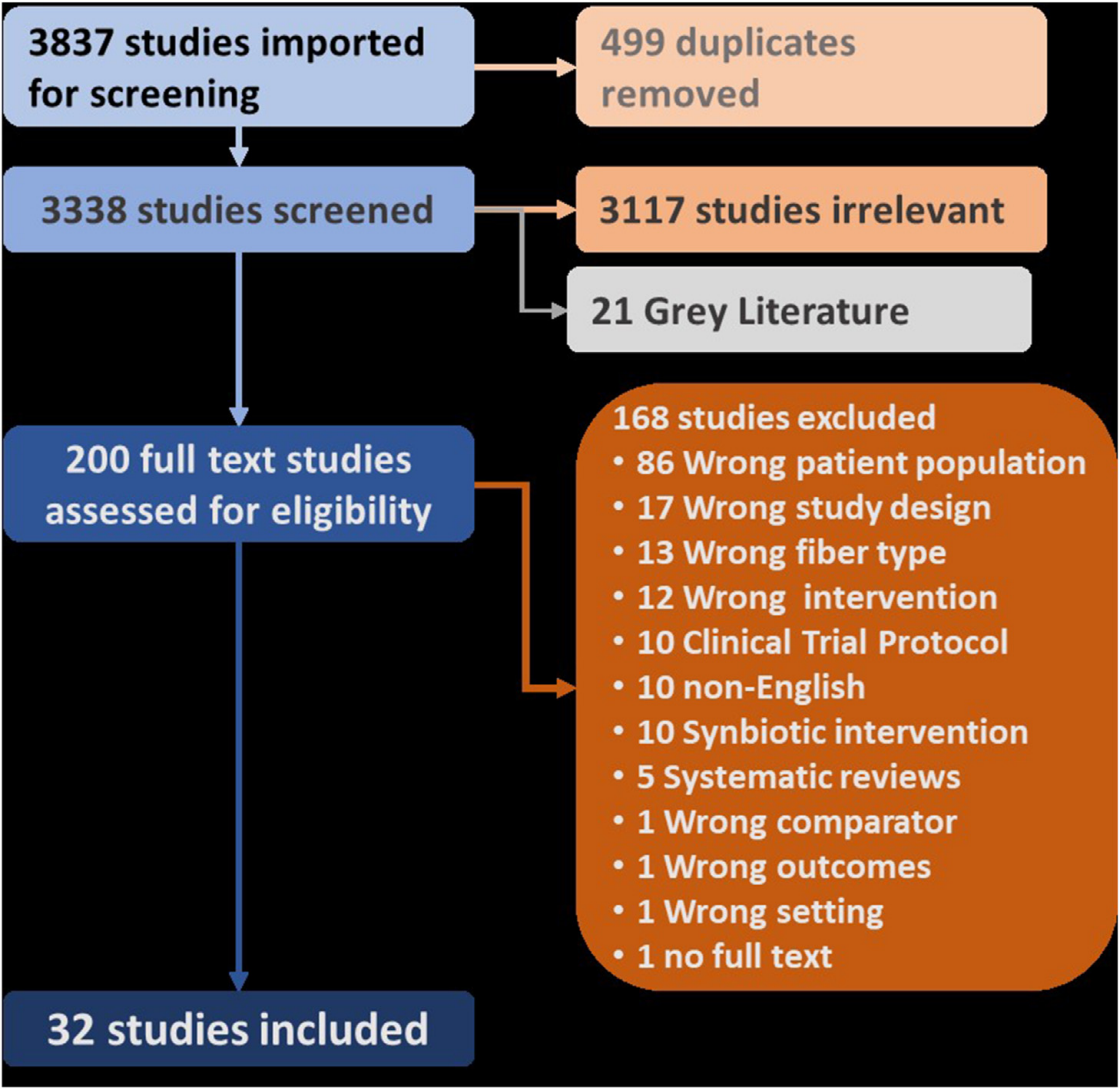
Schematic of the literature search process.

The search and filtering results are shown in Figure 1. A total of 3837 studies were imported for screening. After removing 499 duplicates, 3338 studies were screened at the abstract level. Of these, 21 were categorized as grey literature, and 200 were moved to full-text screening. Of these, 32 studies (list of studies in TABLE 4, TABLE 5, TABLE 8) were deemed eligible for the scoping review. A total of 168 publications were excluded for reasons as shown in Figure 1. The results of the 32 studies were published between 1979 and 2021, as shown in Supplemental Figure 1.

#### Study design summary

Of the 32 studies in the scoping review (TABLE 4, TABLE 5) [[35], [36], [37], [38], [39], [40], [41], [42], [43], [44], [45], [46], [47], [48], [49], [50], [51], [52], [53], [54], [55], [56], [57], [58], [59], [60], [61], [62], [63], [64], [65], [66]], 15 (47%) were parallel RCTs, 15 (47%) crossover RCTs, and 2 (6%) trials with block randomization. Twenty-three studies (72%) were double blinded, 3 (9%) were single blinded, 1 (3%) was nonblinded, and 5 (16%) did not report on this aspect of the study design. The included studies were published between 1979 and 2021, and the research was conducted in Asia (2 studies, 6%), Europe (9 studies, 28%), North America (15 studies, 47%), Central America (1 study, 3%), South America (4 studies, 13%), and the Western Pacific (1 study, 3%). One of these studies was conducted in both United States and Italy.

**TABLE 4:** List of the study country, funding sources, percent male participants, fiber group, and control group interventions for the 32 study articles of the scoping review

First author, reference	Year	PMID	Study country	FS	% Male	Fiber group	Control
Abrams et al. [35]	2005	16087995	United States	G, I	NR	Inulin-type fructans	Maltodextrin
Abrams et al. [36]	2007	17951496	United States	G, I	50.0	Inulin-type fructans	Maltodextrin
Abrams et al. [37]	2007	17719942	United States	G	50.5	Inulin-type fructans	Maltodextrin
Davidson et al. [38]	1996	8604676	United States	NR	NR	Psyllium	Placebo cereal without psyllium
de Bock et al. [39]	2012	22848584	New Zealand	NR	100.0	Psyllium	Potato starch
Dennison et al. [40]	1993	8391569	United States	A, G, I	55.0	Psyllium	Wheat fiber
Drews et al. [41]	1979	474480	United States	G	100.0	Cellulose, hemicellulose, or pectin	Basal diet
Francois et al. [42]	2014	24368315	Belgium	I	64.3	AXOS	Placebo soft drink
Godara et al. [43]	1981	6263076	India	G	0.0	Cellulose	Low fiber diet
Gonzalez et al. [44]	2021	33861390	Mexico	A, I	34.0	Psyllium	Rice flour
Griffin et al. [45]	2002	12088517	United States	G, I	0.0	Inulin-type fructans	Sucrose
Gropper and Acosta [46]	1987	2826563	United States	NR	37.5	Combination of isolated fibers	Placebo supplement
Henao et al. [47]	2018	30317947	Colombia	G	60.0	Beta-glucans	Placebo yogurt
Hume et al. [48]	2017	28228425	Canada	A, G, I	57.1	Inulin-type fructans	Maltodextrin
Kawatra et al. [49]	1993	8387912	India	NR	0.0	Psyllium	Low fiber diet
Liber and Szajewska [50]	2014	25327394	Poland	I	50.5	Inulin-type fructans	Maltodextrin
Lohner et al. [51]	2018	29982534	Hungary	G, I	52.5	Inulin-type fructans	Maltodextrin
Machado et al. [52]	2015	25441591	Brazil	A, G	44.0	Seed gum	Wheat bran
Maki et al. [53]	2003	Not available^1^	United States	NR	72.0	β-glucans	Cereal without β-glucans
Martin et al. [54]	2010	21041813	United States	G, I	0.0	Inulin-type fructans	Cereal without inulin-type fructans
Nicolucci et al. [55]	2017	28596023	Canada	A, G, I	57.1	Inulin-type fructans	Maltodextrin
Ribas et al. [56]	2015	25391814	Brazil	I	51.0	Psyllium	Cellulose
Soldi et al. [57]	2019	30776899	Brazil	G, I	NR	Inulin-type fructans	Maltodextrin
van den Heuvel et al. [58]	1999	10075343	The Netherlands	G, I	100.0	Inulin-type fructans	Sucrose
van den Heuvel et al. [59]	2009	19410973	The Netherlands	I	0.0	Inulin-type fructans	Maltodextrin
Vaz-Tostes et al. [60]	2014	24631386	Brazil	G	53.5	Inulin-type fructans	Control
Vido et al. [61]	1993	8247594	Italy	I	55.0	Glucomannan	Placebo capsule
Vitaglione et al. [62]	2010	20679146	Italy	G	43.8	β-glucans	Placebo meal
Whisner et al. [63]	2014	24848974	United States	I	62.5	SCF	Control diet with no SCF
Whisner et al. [64]	2016	27281813	United States	I	0.0	SCF	Maltodextrin
Williams et al. [65]	1995	8586774	United States	NR	NR	Psyllium	Placebo cereal
Zalewski and Szajewska [66]	2019	31036412	Poland	A, I	46.0	Glucomannan	Maltodextrin

Abbreviations: FS, funding sources; A, academic; G, government; I, Industry; NR, not reported; SCF, soluble corn fiber.

1 https://doi.org/10.1016/S0271-5317(0300178-7).

**TABLE 5:** List of the study design, duration, age range of participants, health status of participants, number of participants analyzed, and fiber group interventions for the 32 study articles of the scoping review

First author, reference	Year	PMID	Type of randomized controlled trial	Study duration (wk)	Age range (y)	Health status	No. of participants analyzed	Fiber group
Abrams et al. [35]	2005	16087995	Parallel	52	9–13	Healthy	98	ITF
Abrams et al. [36]	2007	17951496	Parallel	52	9–13	Healthy	98	ITF
Abrams et al. [37]	2007	17719942	Parallel	52	9–13	Healthy	97	ITF
Davidson et al. [38]	1996	8604676	Crossover	26	6–18	LDL high	25	Psyl
de Bock et al. [39]	2012	22848584	Crossover	14	15–16	OB	45	Psyl
Dennison et al. [40]	1993	8391569	Crossover	12	5–17	LDL high	20	Psyl
Drews et al. [41]	1979	474480	Crossover	3	12–18	Healthy	8	CHP
Francois et al. [42]	2014	24368315	Crossover	9	8–12	Healthy	28	AXOS
Godara et al. [43]	1981	6263076	Crossover	6	16–18	Healthy	9	CLLS
Gonzalez et al. [44]	2021	33861390	Parallel	7	15–19	OB	100	Psyl
Griffin et al. [45]	2002	12088517	Crossover	8	11–14	Healthy	59	ITF
Gropper and Acosta [46]	1987	2826563	Crossover	8	6–12	Healthy	8	CIF
Henao et al. [47]	2018	30317947	Parallel	12	3–5	Healthy	124	βglu
Hume et al. [48]	2017	28228425	Parallel	16	7–12	Healthy	38	ITF
Kawatra et al. [49]	1993	8387912	Crossover	6	16–18	Healthy	11	Psyl
Liber and Szajewska [50]	2014	25327394	Parallel	24	7–18	OB	51	ITF
Lohner et al. [51]	2018	29982534	Parallel	24	3–6	Healthy	219	ITF
Machado et al. [52]	2015	25441591	Parallel	11	10–18^1^	OB	61	SG
Maki et al. [53]	2003	Not available^1^	Crossover	13	6–14	LDL high	18	βglu
Martin et al. [54]	2010	21041813	Crossover	8	11–14	Healthy	14	ITF
Nicolucci et al. [55]	2017	28596023	Parallel	16	7–12	Healthy	38	ITF
Ribas et al. [56]	2015	25391814	Parallel	14	6–19	LDL high	49	Psyl
Soldi et al. [57]	2019	30776899	Parallel	24	3–6	Healthy	209	ITF
van den Heuvel et al. [58]	1999	10075343	Crossover	3	14–16	Healthy	12	ITF
van den Heuvel et al. [59]	2009	19410973	Crossover	7	12–14	Healthy	14	ITF
Vaz-Tostes et al. [60]	2014	24631386	Block randomization	18	2–5	Healthy	89	ITF
Vido et al. [61]	1993	8247594	Block randomization	9	8–14	OB	60	GLM
Vitaglione et al. [62]	2010	20679146	Parallel	Single meal	18	Healthy	16	βglu
Whisner et al. [63]	2014	24848974	Crossover	7	12–15	Healthy	24	SCF
Whisner et al. [64]	2016	27281813	Crossover	8	11–14	Healthy	28	SCF
Williams et al. [65]	1995	8586774	Parallel	12	2–11	LDL high	58	Psyl
Zalewski and Szajewska [66]	2019	31036412	Parallel	12	6–17	OB	81	GLM

AXOS, arabinoxylan oligosaccharide; βglu, β-glucan; CHP, cellulose, hemicellulose, or pectin; CLLS, cellulose; GLM, glucomannan; ITF, inulin-type fructans; Psyl, psyllium; SCF, soluble corn fiber.

1 https://doi.org/10.1016/S0271-5317(0300178-7).

Ten studies (31%) were funded by industry, governmental organizations funded 6 studies (19%), academic centers funded 4 studies (12%), and 12 studies (38%) had mixed sources of funding (Table 4).

The duration of the studies ranged from an acute study of a single meal in 1 day [62] to 52 wk, with 59% of studies ranging from 8 to 12 wk, and 25% of studies being 18 wk or longer as summarized in Table 5 and as also shown in Supplemental Figure 2.

### Study population summary

The number of participants in these studies ranged from 8 to 219 with age ranges from 2 to 18 y. The age categorization varied across studies (Table 6), with research on adolescents and child/early teens being clearly defined in 12 studies respectively. Of the 32 studies, BMI of the populations studied ranged from 23 to 34 kg/m^2^, with 6 studies (19%) [39,44,50,52,61,66] conducted on children with overweight or obesity (BMI ≥ 25 kg/m^2^). Five studies [38,40,44,53,56] were in children reported with either borderline high to high LDL concentration. The baseline LDL values in these studies ranged from 90 to 158 mg/dL. The American Academy of Pediatrics Guidelines for the recommended concentrations of LDL in children state that a value of <100 mg/dL is desirable, 100–129 mg/dL is elevated, and >130 mg/dL represents a high LDL concentration [67]. Based on these criteria, the children in these studies, on average, ranged from elevated to high LDL cholesterol concentrations.

**TABLE 6:** Summary of the number of studies per age range

Age range of participants enrolled (y)	Number of studies
Adolescent (13–18)	12
Child and early teen (6–13)	12
Child and adolescent (6–18)	4
Preschool (1–5)	4
Preschool and child (1–8)	1

### Study intervention summary

Table 7 summarizes the types of fiber that were administered in the intervention arm and the broad categorization of fiber types. The distribution is as follows: inulin-type fructans (ITFs) (13 studies); psyllium (7 studies); β-glucans (3 studies); glucomannan (2 studies); soluble corn fiber (2 studies); cellulose (1 study) or hemicellulose gums (1 study); and arabinoxylan oligosaccharides, combination of isolated fiber, and seed gum (flaxseed; 1 study each).

**TABLE 7:** Categorization of the fiber intervention into broad fiber groupings with corresponding number of studies captured per group

Fiber intervention	Fiber group	Number of individual studies
Oligofructose	Inulin-type fructans	3
Fructo-oligosaccharides	Inulin-type fructans	2
Inulin-type fructan	Inulin-type fructans	2
Oligofructose-enriched inulin	Inulin-type fructans	2
Fructans	Inulin-type fructans	1
Inulin-type fructans product with shorter and longer fructan chains	Inulin-type fructans	1
Short-chain fructo-oligosaccharides	Inulin-type fructans	1
Synergy (Orafti)	Inulin-type fructans	1
Arabinoxylan oligosaccharides	Other fructans	1
Psyllium	Psyllium	5
Isabgol	Psyllium	1
Plantago psyllium	Psyllium	1
Glucomannan	Glucomannan	2
β-Glucans	β-Glucans	2
Barley β-glucan	β-Glucans	1
Cellulose	Cellulose	1
Hemicellulose	Hemicellulose	1
Brown flaxseed	Seed gum	1
Soluble corn fiber	Soluble corn fiber	1
Soluble maize fiber	Soluble corn fiber	1
Combination of isolated fibers	Corn bran, wheat flour, wheat bran, oat flakes, and corn germ meal	1

**FIGURE 2:**
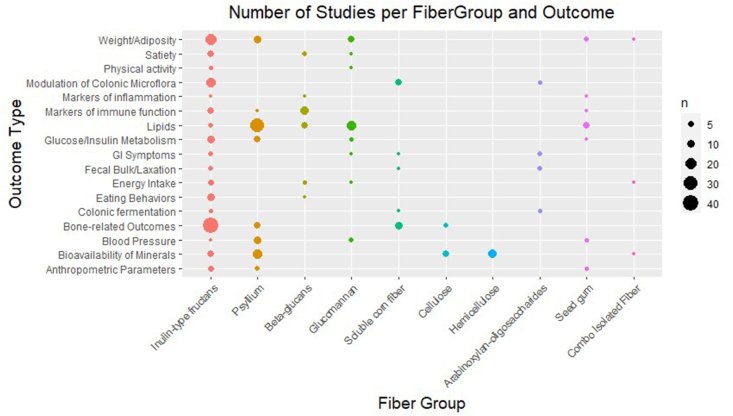
Number of studies per fiber group and outcome.

**FIGURE 3:**
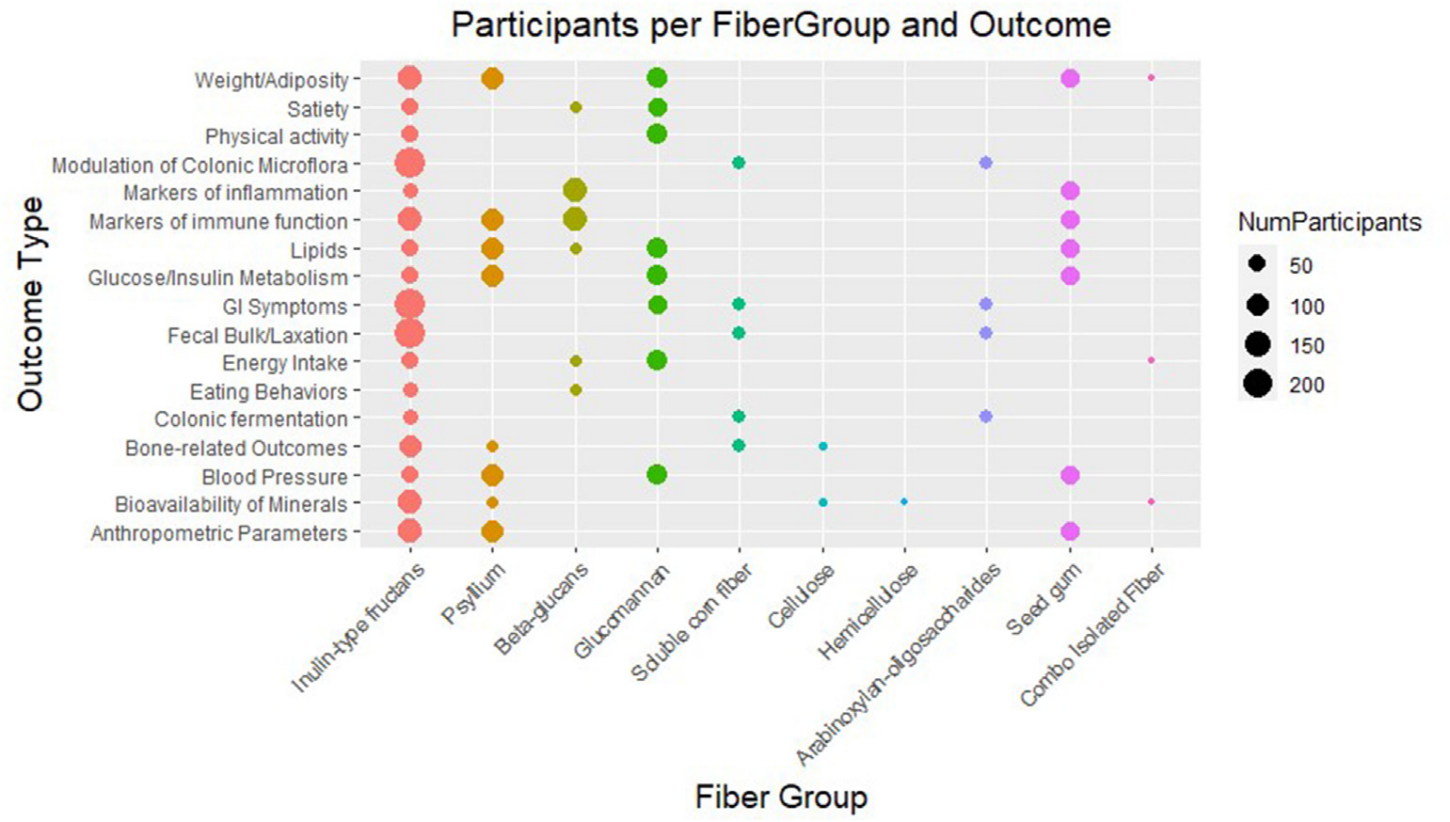
Number of participants in the studies categorized by fiber group in the x axis and reported outcomes in the y axis.

Figure 2 plots the number of studies per fiber group and outcome with the data points sized according to the number of studies. Figure 3 shows the number of participants per study categorized by fiber group and reported outcomes.

### Dosage of fibers summary

For ITFs, the fiber dosage ranged from 2.4 to 15.0 g/d. Psyllium dosages ranged from 6.0 to 25.0 g/d. β-Glucan dosage ranged from 0.35 to 6.0 g/d. Of the studies involving glucomannan, the dosage ranged from 2.0 to 3.0 g/d. For soluble corn fiber, the intervention dosage ranged from 10.0 to 20.0 g/d. For the combination of isolated fiber, the dosage was 5.0 g/d. For cellulose-fiber, the dosage was 21 g/d, and for the modified cellulose-based gum, it was 14 g/d. In the study involving seed gum, the intervention dosage was 28.0 g/d.

### Outcome summary

The number of studies per outcome group is summarized in Table 2, and Table 8 reports on the total outcomes reported captured in each of the publications included in this scoping review. Of note, only 1 study [55] simultaneously measured the modulation of the gut microbiota in relation to change in cardiometabolic risk factors. For each outcome, we provide a summary and note where the effect of the intervention was statistically significant different to the control.

**TABLE 8:**
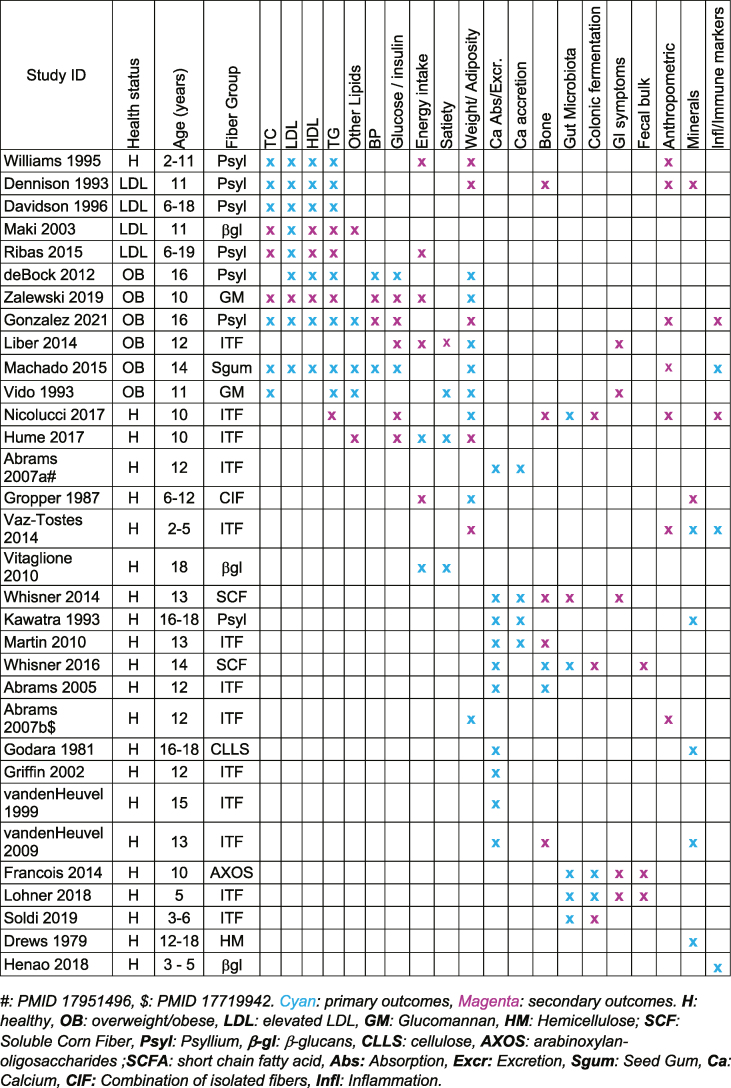
Summary of outcomes reported in the included studies.

### CVD-related outcomes

#### Total and LDL cholesterol

Ten studies measured the effect of a fiber intervention on total and/or LDL cholesterol [38,40,44,53,56,65,66]. Six studies administered psyllium, 1 study administered β-glucans, 2 provided glucomannan, and 1 study had seed gum as the intervention. A significant decrease in total cholesterol concentrations was observed with 3 of the 6 studies involving psyllium [38,56,65]. Four studies involving psyllium [38,39,56,65] reported a significant decrease in LDL cholesterol concentrations. The β-glucan study [53] also reported a significant decrease in LDL cholesterol concentrations. Inconsistent findings were observed with glucomannan with a significant decrease in total and LDL cholesterol concentrations observed at 12 wk but not at 24 wk [66]. One study reported outcomes for very LDL, LDL-1, LDL-2, and small-density LDL cholesterol [44], of which a significant decrease was only reported for small-density LDL in the intervention arm with psyllium.

#### Other markers of lipid metabolism (HDL, TG, and apolipoproteins)

Thirteen studies [44,50,52,55,61,65,66] reported data on markers of lipid metabolism other than total cholesterol and LDL outcomes, in interventions that administered ITFs, psyllium, β-glucans, glucomannan, or seed gum as the intervention. Changes in HDL, TG, adiponectin, non–HDL cholesterol, α-lipoprotein, apolipoprotein A, apolipoprotein A1, apolipoprotein B, β-lipoprotein, and pre–β lipoprotein [61] were reported. In the 6 studies involving psyllium, 1 study [40] reported a significant decrease in TG concentrations. One of the studies using ITF as the intervention [48] showed a significant increase in adiponectin. None of the other studies reported significant changes in the lipids listed.

#### Blood pressure

Five studies [44,50,52,66] examined the effect of the intervention on blood pressure outcomes. No change in blood pressure was reported for ITFs [50], psyllium [44], or seed gum [52]. One study that involves glucomannan [66] reported a significant increase in systolic and diastolic pressure in the 12-wk short-term intervention and a sustained decrease in diastolic but not systolic blood pressure at 24 wk.

#### Glucose and insulin metabolism

Seven studies [39,44,48,50,55,66] examined the effect of the fiber intervention on markers of glucose and insulin metabolism—such as glucose, insulin, fasting glucose, fasting insulin, insulin resistance or insulin sensitivity, ghrelin, and glucagon-like peptide-1. No effect was observed in the change of glucose and insulin metabolism with ITF [48,55], psyllium [39,44], glucomannan [66], or seed gum [52]. One study [48] showed an increase in fasting ghrelin concentrations.

#### Fecal bulk/laxation

Three studies examined the effect of ITFs, arabinoxylan oligosaccharides (AXOS), or soluble corn fiber on fecal bulk/laxation outcomes [42,51,64]. For ITF, significant improvement in stool consistency for ≤24 wk was reported [51]. No change in these outcomes was observed for soluble corn fiber [64] or AXOS [42].

#### Energy intake, satiety, and eating behaviors

Six studies [48,50,65,66] considered the effect of the intervention on energy intake using ITFs, β-glucans or glucomannan, as the intervention. No change in energy intake was reported. One study reported a decrease in prospective food consumption before breakfast [48].

Three studies [48,61,62] reported on satiety outcomes, using ITFs, β-glucans, or glucomannan, as the intervention. A significant increase in satiety was reported for ITFs [48] and β-glucans [62]. Other eating behavior outcomes (desire to eat or drink, food responsiveness, food fussiness, enjoyment of food, and emotional eating) were not significantly different between the intervention arm and the control arm. The desire to eat was reduced significantly relative to control in another study for the smaller load of the β-glucan intervention [62].

#### Weight/adiposity

Thirteen studies examined the effect of ITFs, psyllium, glucomannan, combination of isolated fiber, or seed gum on change in weight or adiposity [36,39,40,44,46,48,50,52,55,60,61,65,66]. Of the 7 studies reporting on BMI [36,39,44,52,55,60,66], 1 study [36] reported a significantly smaller increase of BMI in the ITF arm, 1 study [55] showed no change in the ITF arm during an increase in the control arm, and 2 studies [44,60] showed a decrease of BMI within the intervention arm (ITF and psyllium, respectively) and did not report between-arm results. Glucomannan [66], psyllium [39], and seed gum [52] interventions did not show a change in BMI. Three studies reporting on BMI *z*-scores showed a decrease relative to control [48,50], and 1 study [36] showed a smaller increase in the ITF arm relative to the control arm.

Of the 10 studies [36,39,40,46,50,52,55,60,61,65] that reported on weight as an outcome, 1 study showed a significantly slower weight gain in the ITF arm than that in control [55]. This study also showed a decrease in body weight *z*-score. Weight gain in the study by Abrams et al. [36] was less of an increase than in the control arm. All other studies did not show a significant change between the arms.

Six studies reported on body fat outcomes [36,39,50,55,66]. Two studies on ITF intervention showed either a smaller increase in total fat mass [36] or a decrease [55]. The latter study also reported a reduction in percentage trunk fat and android fat. One study [39] showed a reduction in android-to-gynoid fat ratio. All other studies did not see a significant change in body fat between the arms.

#### Anthropometric parameters

Seven studies [36,40,44,52,55,60,65] reported no change in other anthropometric outcomes (waist circumference, waist-iliac crest, waist-umbilicus, waist:hip ratio, height, and growth velocity), using ITFs, psyllium, or seed gum as the intervention.

### Bone-related outcomes (including calcium absorption and retention)

Twelve studies [35,37,40,43,45,49,54,55,58,59,63,64] examined the effect of fiber on bone-related outcomes, with ITFs [35,37,45,54,58,59], psyllium [40,49], soluble corn fiber [63,64], or cellulose [43] as the intervention. Three of the 6 studies involving ITFs [35,37,57] observed a significant increase in calcium absorption. Soluble corn fiber increased calcium absorption in 1 study [64]. By contrast, a decrease of calcium absorption and retention were observed for psyllium [49] and cellulose [43]. Three studies [37,45,49] reported on net calcium balance. One study [49] showed less of increase in the psyllium arm relative to control, whereas the other 2 studies [37,45] showed significantly higher calcium balance.

One study showed an increase in BMD and whole-body bone mineral content [35], whereas another showed no change in BMD [55]. Bone resorption increased in an ITF intervention [59] as seen by the increase in pyridinoline-to-deoxypyridinoline ratio, although the individual concentrations of pyridinoline and deoxypyridinoline did not show significant changes between arms.

Serum calcium was shown to be reduced in 2 studies [43,49] and did not change in 1 [40]. None of the other bone-related markers [parathyroid hormone, 25(OH) vitamin D, vitamin D index, vitamin D–binding protein, and other bone biomarkers] showed changes for the fiber intervention in any of the studies reporting bone-related outcomes.

### Gut-related outcomes

#### Modulation of colonic microflora

Six studies [42,51,55,57,63,64] reported on the modulation of colonic microflora using ITFs, AXOS, or soluble corn fiber as the intervention. Three studies with ITF as intervention [51,55,57] observed a significant increase in the relative abundance of bifidobacteria and/or lactobacilli in the intervention arm relative to the control arm. Two studies of soluble corn fiber [63,64] reported an increase in proportion of bacteria of the phylum Bacteroidetes and a reduction in the average proportion of phylum Firmicutes, in feces. One of the 2 studies that involves soluble corn fiber [64] also reported a significant increase in bifidobacteria. A significant change in microbiota composition was reported for ITF [42,51,55,57] and soluble corn fiber interventions [63,64]. Four studies reported on fecal pH outcomes [42,51,57,64] of which it was found to be lower relative to control after soluble corn fiber intervention [64].

#### Colonic fermentation/short-chain fatty acid production

Five studies [42,51,[55], [57],^64^] reported on colonic fermentation or short-chain fatty acid production, using ITFs (3 studies), AXOS (1 study), or soluble corn fiber (1 study). One study of ITFs [42] observed a significant decrease in branched-chain fatty acids. Single-chain fatty acids did not change after ITF or AXOS intervention [42,64]. Three studies reported on fecal pH [51,57,64], and only 1 of them showed a significant decrease [64].

#### GI symptoms

GI symptoms included abdominal pain or discomfort, flatulence, urge to vomit, constipation, and diarrhea. Five studies [42,51,55,61,63] reported no change in GI symptoms using ITFs [50,51], AXOS [42], glucomannan [61], or soluble corn fiber [63].

#### Bioavailability of minerals

Eight studies [40,41,43,46,49,54,59,60] included measures on the bioavailability of minerals with ITFs, psyllium, cellulose, hemicellulose, or combination of isolated fiber as the intervention. In studies involving ITFs, 1 study [59] reported a significant increase in long-term (36 d) magnesium absorption. In 2 studies with psyllium as the intervention, 1 [49] reported a significant lower increase in iron balance and phosphorous balance in the psyllium arm relative to the control. Both the psyllium studies [40,49] reported on serum iron that was reduced in the intervention arm in one study [49] whereas being significantly higher in the other [40]. One study consisting of a combination of isolated fiber mixture (see Table 7 for composition) intervention reported no change in bioavailability of mineral outcomes. One study involving cellulose [43] reported a significant decrease in iron and phosphorus absorption and in serum inorganic phosphorus and serum iron. One study with hemicellulose as the intervention [41], reported a decrease in copper, magnesium, and zinc balances. In the same study, no changes were seen in the cellulose and pectin arms for the same outcomes.

#### Markers of inflammation and immune function

Five studies [[44], [47], [52], [55], [60]] reported on inflammation or immune function outcomes, using ITFs, β-glucans, psyllium, or seed gum as the intervention. One study that involved ITFs [60] reported a significant increase in fecal secretory Ig A and a decrease in IL-6 in another [55]. For the β-glucan intervention [47], there was an increase in the numbers of circulating and peripheral CD3^+^, CD4^+^, CD8^+^ cells but no changes in natural killer cells or serum concentrations of secretory IgA or cytokines. One study that involves psyllium [44] reported a significant decrease in IL-6 concentration. No changes were reported for IL-6, C-reactive protein, interferon-γ, IL-10 and TNF-α for the psyllium or seed gum interventions [44,52].

## Discussion

This scoping review identified several isolated fibers that have been examined in relation to a wide range of health outcomes in children. The most studied fibers were the ITFs (41%), followed by psyllium (22%) and β-glucans (9%). The most commonly studied outcomes were cardiometabolic-related (50%) and growth-related (bone-related?) (∼38%) outcomes.

Within the category for ITF, variation existed in the types of fructans administered, including short-chain fructose molecules (2–10 units, for example, oligofructose or short-chain fructo-oligosaccharides) [50,58,59], longer-chain fructans (10–60 units, for example, fructo-oligosaccharides) [50,51,58,60], fructans [[35], [36], [37]] or a combination of fructans, such as oligofructose-enriched inulin [45,46,55], and the product Synergy (Orafti) [37,54], and a combination of short-chain and long-chain inulins [57]. All these are considered prebiotic fibers: “a substrate that is selectively utilized by host micro-organisms conferring a health benefit” [68]. Growing evidence suggests that prebiotics may play a role in mineral absorption [69] and immune function [70] through its influence on the gut microbiota. In this scoping review in children, we identified the effect of fiber on bone-related outcomes in 12 studies [[35], [37],^40^,^43^,[45], [49],[54], [55],^58^,[59], [63], [64]] and markers of inflammation in 5 studies [44,47,52,55,60]. The diversity in fiber types leads to variation in gut microbiota and metabolic-end products, leading to different metabolic response and potential health benefits. The FDA considers modulation in the gut microbiota to be a benefit to health [33,71], thus linking the microbiome-gut effects to health benefits as a high priority in research. Of the 5 studies [42,51,55,57,64] reporting on colonic fermentation or short-chain fatty acid production, only 2 studies reported other health outcomes. One of these studies examined the effect of oligofructose-enriched inulin on modulation of the gut microbiota on body weight changes and changes in markers of systemic inflammation in healthy children with overweight or obesity [58]. Another study [64] examined the effect of soluble corn fiber, a resistant maltodextrin, on microbial diversity and calcium absorption in healthy adolescent females. Further research in children and adolescents, considering the complex role of dietary fibers on the microbiome and its mediated health effects is needed.

With respect to the physiologic characteristics of fiber, the majority of fibers (50%) were soluble, viscous (gel forming), and fermentable. These included AXOS, soluble corn fiber, glucomannan, and β-glucan, whereas psyllium and flaxseed, representing 20% of fibers, are considered soluble, viscous, but are nonfermentable fibers. Only highly viscous soluble fibers (for example, gel-forming fibers) can impact lipid and glucose metabolism to potentially lower cholesterol concentrations [6]. Soluble, nonviscous, readily fermented fibers such as inulin and oligosaccharides have the capacity to improve health via the modulation of the gut microbiome. These fermentable fibers are an emerging area of research, for which most of the RCTs in this scoping review fall under this classification.

Glucose and insulin metabolism were reported for 7 studies, of which only 3 studies reported on fasting glucose outcomes [52,55,66] and only 1 on fasting insulin [55], with the remainder reporting on plasma glucose, insulin, insulin sensitivity, and glucose-dependent insulinotropic polypeptide. The majority of interventions did not observe an effect of the fiber on measures of glucose metabolism, which may be partly attributed to better glycemic control in this age demographic, because of insufficient fiber dose to promote glucose reduction [52], or because the fiber administered is not effective in impacting glucose metabolism (for example, ITF) [72]. Regarding body weight, 5 of the 13 studies (38%) showed significantly slower gain in weight [36,55] or fat mass [36,39,48,50,55]. No changes were observed in anthropometric outcomes other than weight or adiposity. This finding was not unexpected given most of these studies recorded isocaloric content or caloric content was not reported [39] and/or of short duration (<24 wk) [39,48,50,55]. It is also worth noting that measuring weight changes in adolescents is subject to measurement error because of growth spurts and change in body composition [73]. Of the 13 studies on body weight/fat mass, ∼40% were conducted in adolescents who may be experiencing physiologic changes associated with puberty.

The effect of a variety of prebiotic fibers (ITF, AXOS, and soluble corn fiber) on colonic fermentation or short-chain fatty acid production, a by-product of fermentation, was reported in 5 RCTs with study duration ranging from 8 to 24 wk (Table 5) [42,51,55,57,64]. Most of these interventions showed a significant change in the microbiota composition or microbial diversity. Only 1 study that examined the effect of fiber on gut microbiota also considered other risk factors, such as weight, adiposity, inflammatory markers, fasting glucose and insulin, fecal markers, and BMD [55].

The studies reported no changes in GI symptoms or eating behaviors. No consistent changes were seen for inflammatory markers or markers of immune function. One of the 3 studies (33%) showed improved fecal bulk, and no studies reported on colonic transit time in this population.

### Evidence-rich areas and evidence gaps

The most studied isolated fiber group was ITFs (15 studies), followed by psyllium (7 studies) and β-glucans (3 studies). All other fiber groups had <3 studies each. The most studied outcomes were lipid outcomes (HDL, TG, and lipoproteins) with 13 studies, followed by weight/adiposity (12 studies) and bone-related outcomes (10 studies). Some of the less-studied outcomes related to fecal bulk and laxation (∼3 studies). Postprandial glycemia/insulinemia or colonic transit time were not studied at all in this population. Considering the prevalence of constipation or the rise of metabolic syndromes in younger populations [74], these outcomes may be worth additional investigation. Dietary fibers have the potential to negatively impact health by impairing the absorption of minerals in the small intestine, which may, in turn, affect bone health. However, the evidence from clinical trials in children considering how isolated fibers impact mineral absorption or mineral balance is limited. In this scoping review, 3 of the 6 studies involving studies involving ITFs [35,37,58] observed a significant increase in calcium absorption and 1 observed a positive impact on magnesium absorption [58]. The overall scientific evidence, as reviewed by the FDA, supports that ITF have a beneficial physiologic effect on BMD and absorption of calcium [33]. For other dietary fibers, the strength of the evidence to support a bone-related health impact is limited, and our review points to the need for more research to examine the impact of fiber on mineral absorption and status-related biomarkers with longer intervention time frames. Currently, the FDA [33] has identified several isolated or synthetic fibers as meeting the fiber definition, attributing them to ≥1 physiologic health benefit. Most of the research in this area has been on evidence from adult populations, with outcomes relevant to adult health, such as lowering blood pressure, lipids, or glucose. Recognizing that intervention studies in children and teens are challenging, in both practical and ethical considerations, strong evidence demonstrating that isolated fibers deliver health benefits in adults is needed before consideration of interventions in children. We eliminated studies containing pea hull fiber, galacto-oligosaccharides, locust bean gum, blueberry husks, rye bran, mango juice by-product, chitosan, sip feeds, methyl cellulose, and synbiotics for various reasons as explained in the Methods section. Thus, when considering dietary recommendations, evidence-based research is needed to enhance our understanding of how isolated dietary fibers influence health outcomes in children. Nonetheless, given the prevalent low intake of fiber, there is a crucial need to promote the consumption of natural dietary fiber from foods. Only 1 RCT in overweight adolescents compared the effects of seed gum fiber from ground flaxseed with those of fiber in wheat bran (control) added to a variety of foods on lipid markers, blood pressure, markers of inflammation, and body composition [52]. More research is needed in studies that explore the impact of natural-fiber rich foods such as prunes, flaxseed, or chia seeds for instance, in comparison to isolated fibers. This comparative design would enhance our understating of their effects in relation to naturally occurring counterparts. In addition, the included studies did not consider in their design age groups that align with how the dietary guidelines are set for kids: 2–3 y, 4–8 y, 9–13 y, and 14–18 y. Hence, interpretation of these data according to the specific age groups was not possible. We see this as a gap in how children’s studies on this topic are structured and would suggest that future studies should be designed with the age groups aligned with those used for dietary guidelines specifically.

### Strengths and limitations

The results from our review inform future areas of research, indicating that there is sufficient evidence available to conduct systematic reviews and meta-analyses of RCTs evaluating the effect of isolated or synthetic fiber on health outcomes in children. The general recommendation [75] is to have a minimum of 3 studies per outcome for a meta-analysis. Based on this rather loose criterion, there are several outcomes for which a systematic review and meta-analysis could be performed, including cardiometabolic factors (lipid, weight, and adiposity outcomes), bone-related outcomes, modulation of colonic microflora, glucose and insulin metabolism, energy intake, fecal bulk/laxation and, satiety, provided other criteria for a robust meta-analysis are met. Based on a somewhat arbitrary but more stringent criterion of ≥7 studies required for a meta-analysis, we note that systematic review and meta-analyses can be performed for 6 outcomes—bone lipid metabolic outcomes, weight/adiposity, bone-related outcomes, bioavailability of minerals, glucose and insulin metabolism, and total and LDL cholesterol. Our review also identified that ITFs and, to a lesser extent, psyllium are well represented in the literature, thus highlighting the importance of future research studying non-ITF fibers. In addition, cardiometabolic and growth/bone-related outcomes are commonly studied, compared with a notable gap in the literature examining the effects of fiber intake on other health outcomes, such as modulation of gut microbiota. In this review, we broadly categorized fiber types (Table 7); however, if sufficient research were available, the categorization of certain fibers could be more refined. For example, ITFs could be classified based on chain length, which is an important consideration as chain length impacts the degree of bacterial growth and gut microbiome diversity, which, subsequently, can impact health.

Scoping reviews, by nature, provide a limited perspective as they do not aim to assess the quality of the studies included; quality assessment is something that would be instead performed in a systematic review [76]. In addition, the dosages of the fiber types reported in the included studies varied widely. An assessment of the equivalencies of doses across fiber types is needed, such that the data can be used appropriately in a meta-analysis.

Although our inclusion criteria focused on populations of healthy children, it did include study populations who were overweight, who were obese, or with elevated cholesterol. The latest evidence reports that over 1 in 5 children in the United States are obese [77] and that >1 in 3 are overweight. Similarly, it is estimated that among United States children aged 12–18 y, elevated LDL cholesterol (≥130 mg/dL) occurs in 6.1% and 3.0% of male and female adolescents, respectively [78]. This review aimed to include a representative sample of children in the United States and, thus, did not exclude populations of overweight or obese children or those with elevated lipid concentrations.

## Conclusions

Our scoping review has identified 32 clinical trials investigating the impact of isolated fibers on health outcomes. Our review demonstrates that there is sufficient evidence associated with cardiometabolic outcomes such as weight, adiposity, lipid concentrations, fasting insulin and glucose metabolism, and gut health to conduct a systematic review and meta-analysis. We also note sufficient evidence for bone-related outcomes such as calcium absorption, vitamin D concentrations, and parathyroid hormone concentrations. Thus, there is enough evidence for several outcomes to warrant a systematic review and meta-analysis of the impact of fiber intake on these outcomes in children. However, evidence gaps have been identified for certain outcomes or certain fiber interventions, which can inform a funding strategy for additional studies.

## Acknowledgments

We thank Elizabeth Zalis for her contributions toward inspection of the articles that formed part of the scoping review results.

### Author contributions

The authors’ responsibilities were as follows – NN: designed the overall research plan, managed the project, and contributed to the manuscript; RM: performed the literature search; LC, EA, AVV, JLL, DT, NN: conducted the literature analysis and data extraction; LC, EA, NN: summarized the results of the data extraction; NM, KA: provided critical input regarding the results and contributed to the discussion; LC, EA, AVV, NM, KS: contributed to the manuscript writing; and all authors: have read and approved the manuscript.

### Conflict of interest

N.M.M. is an unpaid Scientific Advisor for Whole Grains Council and has received a gift-in-kind from P&G. All other authors declare that they have no competing interests. The funders had no role in the design of the study; in the collection, analyses, or interpretation of data; in the writing of the manuscript, or in the decision to publish the results.

### Funding

The Institute for the Advancement of Food and Nutrition Sciences

### Data availability

Data described in the manuscript, code book, and analytic code will be made publicly and freely available without restriction.
