# Supplementary material for: Physiologic Effects of Isolated or Synthetic Dietary Fiber in Children: A Scoping Review
Source: Curr Dev Nutr. 2024 Jan 4;8(2):102074. doi: 10.1016/j.cdnut.2023.102074 (PMC10847057; doi:10.1016/j.cdnut.2023.102074)
Supplement: Multimedia component 1 [file mmc1.docx]

Physiological effects of isolated or synthetic dietary fiber in children: a scoping review*.*

First Author: Linfei Chen

*Supplemental Table S1: Search Strategy in MEDLINE for the impact of isolated and synthetic fiber on children’s health outcomes.*

| **Step** | **Search Terms** | **Number of Articles** |
| --- | --- | --- |
| 1 | Dietary fiber.mp. or exp Dietary Fiber/ | 23489 |
| 2 | ("dietary fibre" or "functional fibre" or "added fibre" or "dietary fiber" or "functional fiber" or "added fiber").mp. | 21358 |
| 3 | 1 or 2 | 24434 |
| 4 | Alginates/ | 12775 |
| 5 | (Alginates or Alphacyclodextrin or Alpha-cyclodextrin or Arabinoxylan or Arabinoxylan-oligosaccharides or AXOS).mp. | 15408 |
| 6 | aleurone.mp. | 886 |
| 7 | beta-Glucans/ | 4523 |
| 8 | ("Barley Bran" or "Barley grain" or Beta-glucans).mp. | 5689 |
| 9 | Carrageenan/ | 8044 |
| 10 | Chitin/ | 7310 |
| 11 | Chitosan/ | 23529 |
| 12 | Cellulose/ | 30552 |
| 13 | (Carrageenan$ or Cellulose or Chitin or Chitosan or "Corn bran").mp. | 121807 |
| 14 | Fructans/ | 1170 |
| 15 | (Fructan$ or Fructooligosaccharide$ or Fructo-oligosaccharide$ or "short chain fructo-oligosaccharide" or "short chain fructooligosaccharide").mp. | 3306 |
| 16 | (Galactooligosaccharide$ or Gum$ or Galactomannan or Arabinogalactan).mp. | 23838 |
| 17 | (Hemicellulose$ or "Hydroxypropyl methylcellulose$" or Hydroxypropylmethylcellulose$).mp. | 7960 |
| 18 | Inulin/ | 7104 |
| 19 | (Inulin or "wheat dextrin" or dextrin$ or "resistant wheat starch$" or "wheat starch$" or "resistant starch$").mp. | 15627 |
| 20 | (sterculia or "Konjac mannan$").mp. | 206 |
| 21 | (Legume$ or "bean fiber" or Lignin or "Locust bean" or Methylcellulose).mp. | 37341 |
| 22 | ("Oat Bran" or Oligofructose or "Okra adj2 gum" or "guar adj2 gum").mp. | 899 |
| 23 | psyllium/ | 690 |
| 24 | Pectins/ | 6194 |
| 25 | ("Pea fiber" or "pea hull" or Pectin or Polydextrose or "Potato fiber" or Psyllium or Ispaghula).mp. | 8852 |
| 26 | Karaya Gum/ | 127 |
| 27 | Gum Arabic/ | 843 |
| 28 | ("gum adj2 arabic" or "high amylose starch" or "karaya adj2 gum" or "carob adj2 gum").mp. | 147 |
| 29 | Xylans/ | 3375 |
| 30 | ("Rice Bran" or "Rye fibre" or "Rye fiber" or "Rye Bran" or "Soluble corn fiber" or "Soy fiber" or "Sugar Beet" or sugarbeet or "sugar beet fib$" or "sugarbeet fib$ or Sugar cane fib$ or Tara gum or Wheat Bran or Xanthan gum" or Xylans or Xyloglucans).mp. | 7643 |
| 31 | high amylase starch$.mp. | 1 |
| 32 | "chemically modified starch$".mp. | 38 |
| 33 | ("resistant maltodextrin" or resistant dextrin$).mp. | 77 |
| 34 | (nondigestible carbohydrate$ or non-digestible carbohydrate$).mp. | 225 |
| 35 | 4 or 5 or 6 or 7 or 8 or 9 or 10 or 11 or 12 or 13 or 14 or 15 or 16 or 17 or 18 or 19 or 20 or 21 or 22 or 23 or 24 or 25 or 26 or 27 or 28 or 29 or 30 or 31 or 32 or 33 or 34 | 222727 |
| 36 | (Benefiber or Citrucel or FiberChoice or Fibersure or Hi-Maize or Konsyl or Fibersol or Metamucil or Normacol).tw. | 111 |
| 37 | (Actilight or Meiologio or NutraFlora or neosugar or Normafib or Orafti or Synergy1).tw. | 69 |
| 38 | (Litesse or Nutriose or Novelose or Fibersym).tw. | 55 |
| 39 | (actistar or BranaVita or Fibrulose or GrainWise or Oliggo-Fiber or Oliggofiber or Fibruline or "metamucil clear and natural" or "metamucil clear & natural" or naturaflora or normafibe or "orafti inulin" or "beneo synergy 1" or "beneo synergy" or "novelose 330" or novelose).tw. | 23 |
| 40 | 36 or 37 or 38 or 39 | 240 |
| 41 | ((isolate$ adj3 fiber) or (isolate$ adj3 dietary fiber)).mp. | 383 |
| 42 | ((synth$ adj3 fiber) or (synth$ adj3 dietary fiber)).mp. | 480 |
| 43 | ((isolate$ adj3 fibre) or (isolate$ adj3 dietary fibre)).mp. | 134 |
| 44 | ((synth$ adj3 fibre) or (synth$ adj3 dietary fibre)).mp. | 108 |
| 45 | (functional adj (fiber or fibre)).mp. | 60 |
| 46 | 41 or 42 or 43 or 44 or 45 | 1157 |
| 47 | exp “diet, food, and nutrition”/ | 1816523 |
| 48 | diet therapy.fs. | 54224 |
| 49 | (diet$ or nutrition$).tw. | 723012 |
| 50 | 47 or 48 or 49 | 2121034 |
| 51 | 3 or 35 or 40 or 46 | 240741 |
| 52 | 50 and 51 | 74163 |
| 53 | (child or children or adolescen$ or infan$ or toddler$ or teen$ or young child$ or preschool$ or pre-school$ or pre school$ or pediatric$ or paediatric$ or youth$ or juvenile$ or school$).tw. | 1982203 |
| 54 | 52 and 53 | 3653 |
| 55 | exp animals/ not humans.sh. | 4895741 |
| 56 | 54 not 55 | 3394 |
| 57 | (randomized controlled trial or controlled clinical trial).pt. or randomized.ab. or placebo.ab. or drug therapy.fs. or randomly.ab. or trial.ab. or groups.ab. or intervention$.ab. | 5254120 |
| 58 | exp clinical trial/ | 911262 |
| 59 | 57 or 58 | 5375423 |
| 61 | 56 and 59 | 1617 |
| 62 | 56 not 60 | 1777 |

*Supplemental* *Figure S1: Timeline of publications of the studies per fiber group.*

***
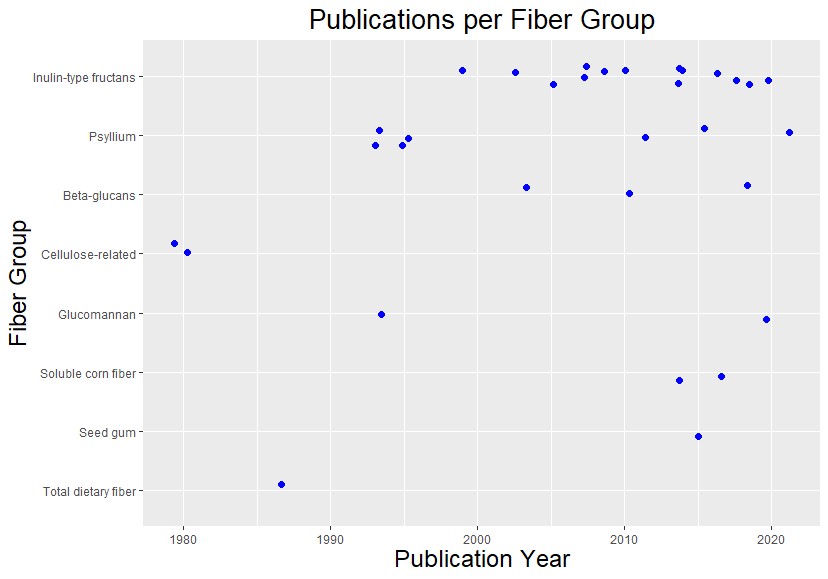
***

*Supplemental Figure S2: Summary of the number of studies per study duration.*

**
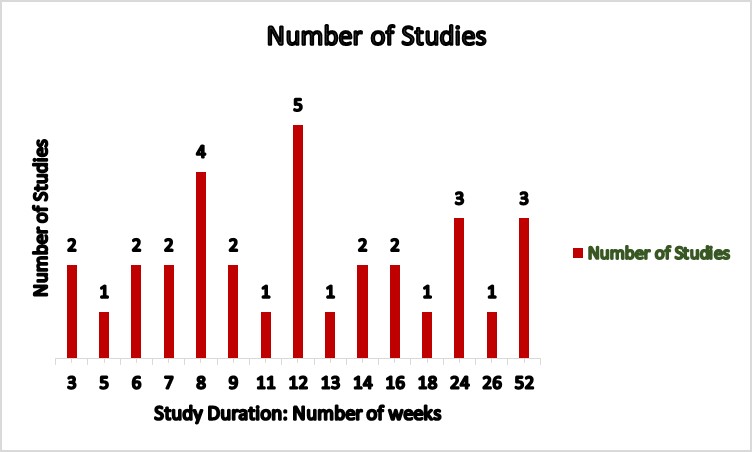
**
